# Fecal Loading at Caecum as a New Radiological Sign for Diagnosing Acute Appendicitis

**DOI:** 10.7759/cureus.20903

**Published:** 2022-01-03

**Authors:** Nazia Haider, Zahid Mehmood, Vijay Kumar, Marium Imran, Moiz Ahmed

**Affiliations:** 1 Department of Surgery, Jinnah Postgraduate Medical Centre, Karachi, PAK; 2 Department of Medicine, Jinnah Postgraduate Medical Centre, Karachi, PAK

**Keywords:** gastroentrology, abdominal radiography, x-rays, fecal loading, acute appendicitis

## Abstract

Background

The present study aimed to address the importance of a new radiological sign - the presence of fecal loading at the caecum - for the diagnosis of acute appendicitis.

Methodology

A cross-sectional study was conducted at the Department of General Surgery, Jinnah Postgraduate Medical Centre, Karachi from January 2020 to June 2020. Patients who presented in the emergency with acute pain at the right iliac fossa fulfilling the criteria of acute appendicitis (AA) according to the Alvarado scoring system, and were planned for appendectomy were included. Before surgery plain abdominal radiographs were taken in anteroposterior view in the supine position and were evaluated for the presence of fecal loading at the caecum. After that all patients underwent surgery and radiologic findings were correlated with histopathologic findings.

Results

The mean age of patients was 32.19±7.34 years. There were 83 (55.3%) male and 67 (44.7%) female patients. Out of 150, there were 144 (96.0%) patients in whom fecal loading in the caecum was diagnosed on plain radiographs. On histopathology reporting, acute appendicitis was diagnosed in 143 (95.3%) patients. Regarding accuracy, fecal loading at the caecum was found to have a sensitivity of 98.6%, specificity of 83.3%, a positive predictive value of 99.3%, and a negative predictive value of 71.4%.

Conclusion

According to the results of the present study and existing literature, we suggest using fecal loading at the caecum along with a clinical scoring system for the diagnosis of acute appendicitis. As per our findings, fecal loading at the caecum is a valuable sign on plain abdominal radiograph for the diagnosis of AA. It has a sensitivity of 98.6% and a specificity of 83.3%. This sign typically becomes undetectable after an appendectomy. It will help to improve the accuracy of diagnosis of acute appendicitis, and hence will reduce the chances of negative appendectomy.

## Introduction

Acute abdomen is a common emergency accounting for 7 to 10% of all emergency department admissions [1]. Acute appendicitis is the commonest cause of acute abdomen and is one of the most frequent diagnoses in young people visiting emergency departments [2]. The incidence of acute appendicitis has been declining since the 1940s in the modern world. The reported incidence from the developed world is 5.7 to 50 patients/100,000 population/year, while age 10 years to 30 years is reported to peak age of developing acute appendicitis [3,4]. Incidence is 10 times higher in developing countries and is increasing [5]. Acute appendicitis incidence is also affected by seasonal variations, incidence in Pakistan is higher in the summer season as compared to the winter season [6]. 

Acute appendicitis can lead to perforation in about 16%-40% of cases, with a higher incidence in older age (55% to 70%) as compared to the younger age due to delay in making the correct diagnosis [7]. The diagnosis of acute appendicitis is often challenging and involves a series of clinical, laboratory, and radiological investigations. To improve the diagnosis and to enhance accuracy to prevent false diagnosis various scoring systems and diagnostic criteria have been developed to determine the diagnosis of acute appendicitis [8,9].

Plain radiographs are easily available radiology imaging readily available in all centers. Many radiographic findings have been found to be correlated with the presence of acute appendicitis, these include, (1) fluid levels localize to the caecum and terminal ileum, indicating inflammation in the right lower quadrant, (2) local ileus with gas in the caecum, ascending colon, and terminal ileum, (3) appendicoliths, (4) gas-filled appendix, (5) dilated small bowel loops in lower quadrant, (6) loss of right psoas outline, (7) strip of free air along the right paracolic gutter, delineating the lower border of the liver (perforated appendix), (8) increase soft tissue density of right lower quadrant, and (9) fecal loading in the caecum [10].

The aim of this study is to determine the role of fecal loading at the caecum in the diagnosis of acute appendicitis. We could not understand the pathophysiology to answer why appendicitis could lead to fecal loading in the caecum. However, we believe it is worth noting that this sign may be related to the local ileus of the caecum with stool in the presence of acute inflammatory conditions. This is one of the few studies conducted to explore fecal loading as a radiological sign for acute appendicitis.

## Materials and methods

A cross-sectional study was conducted between February 2020 to December 2020 at the department of surgery, Jinnah Postgraduate Medical Centre, Karachi. A non-probability convenience technique was employed to include participants in the study. A sample size of 150 was calculated using OpenEpi (www.openepi.com), by keeping the confidence level of 95%, a margin of error of 4.48%, and the lifetime incidence of acute appendicitis as 8.6% [11]. 

Patients who presented with right iliac fossa pain fulfilling the criteria of acute appendicitis according to the Alvarado scoring system, and were planned for appendectomy were included. Institutional Review Board approval (F2-81/2020-GENL/48527/JPMC) was obtained prior to the study. A written and informed consent was taken from the patients after narrating the objectives of the study along with any risk associated with it.

Before surgery, plain radiographs were taken and were evaluated for the presence of fecal loading at the caecum. After that all patients underwent surgery and radiologic findings were correlated with histopathologic findings. For the patients in whom caecum loading was found, the radiographs were taken again on the next day after surgery to determine whether the fecal loading was also present on the next day after surgery or not. All data was documented in a preformed proforma. 

Data were analyzed using SPSS 25 software (IBM Corp., Armonk, USA). All continuous variables were presented as mean and standard deviation while all categorical variables were presented as frequency and percentage. The 2×2 contingency table was formulated to determine the sensitivity, specificity, positive predictive value, and negative predictive value of fecal loading in the caecum on plain radiograph taking histopathology as the gold standard.

## Results

The mean age of patients was 32.19 ± 7.34 years. There were 83 (55.3%) male and 67 (44.7%) female patients. The mean duration of pain was <12 hours in 13 patients, 12-24 hours in 94 patients, 24 to 72 hours in 36 patients, and >72 hours in seven patients. Out of 150, there were 144 (96.0%) patients in whom fecal loading at the caecum was diagnosed on plain radiographs. On histopathology reporting, acute appendicitis was diagnosed in 143 (95.3%) patients. Regarding accuracy, fecal loading at the caecum was found to have a sensitivity of 98.6%, specificity of 83.3%, a positive predictive value of 99.3%, and a negative predictive value of 71.4% (Table 1).

**Table 1:** Baseline characteristics of study participants

Age (mean ± SD) (years)	19.26 ± 13.48
Age Groups	
<5 years	1 (0.67%)
5 to 10 years	12 (8.00%)
10 to 15 years	42 (28.00%)
16 to 20 years	36 (24.00%)
Above 20 years	59 (39.33%)
Gender	
Female	63 (42.00%)
Male	87 (58.00%)
Symptoms	
Pain (Right Iliac Fossa)	67 (44.67%)
Pain (Right Iliac Fossa), Vomiting and Fever	60 (40.00%)
Generalized Abdominal Pain	10 (6.67%)
Generalized Abdominal Pain and Burning Micturition	12 (8.00%)
Others	1 (0.67%)

Out of the 143 cases of acute appendicitis, 142 patients had fecal loading at the caecum while one case did not present with fecal loading (Table 2). 

**Table 2:** Diagnostic accuracy of fecal loading at caecum for diagnosis of acute appendicitis

Fecal loading at Caecum		Acute Appendicitis on Histopathology		Total
		Yes	No	
Positivity	Yes	142	01	143
	No	01	06	07
Total		143	07	150

The sensitivity, specificity, positive predictive value, and negative predictive value for fecal loading at the caecum are presented in Table 3.

**Table 3:** Indices for diagnostic accuracy

Indices for Diagnostic Accuracy	Value	95% Confidence Interval
Sensitivity	98.61%	95.07% to 99.83%
Specificity	83.33%	35.88% to 99.58%
Positive Likelihood Ratio	5.92	0.99 to 35.41
Negative Likelihood Ratio	0.02	0.00 to 0.07
Positive Predictive Value	30.81%	6.93% to 72.72%
Negative Predictive Value	99.87%	99.48% to 99.97%
Accuracy	84.40%	77.59% to 89.81%

## Discussion

Acute appendicitis (AA) is the commonest indication of emergency surgery in patients presenting in emergency departments. Usually, the presentation is seasonal, the majority of patients (up to 40%) present in the summer season in Pakistan [6]. The incidence occurs at a young age, >90% of patients present before the age of 50 years [12]. 

The morbidity and mortality associated with acute appendicitis are very low if it is diagnosed in early time and hence operated accordingly. If the diagnosis is delayed, the risk of perforation is also increased [13]. In cases of perforated appendicitis, the mortality rate is at least 5% which is higher than in non-perforated appendicitis [14]. 

However, in suspicion cases where there is suspicion of other underlying diseases, imaging investigations are done to confirm the diagnosis. Plain X-rays are the first-line imaging investigation in many diseases including acute appendicitis [15]. The first large-scale investigation on the utility of plain X-rays for evaluation of acute appendicitis was conducted by Steinert et al. The authors divided the X-rays findings into five categories of the probability of acute appendicitis. The authors reported that in up to 72% of patients of AA, there is evidence of AA of two or more signs on plain radiographs. And in patients with gangrene or perforation, positive X-rays findings are present in up to 72% of cases [16]. 

In 2005, Petroianu et al. reported the use of plain X-rays for diagnosis of acute appendicitis, the authors reported that the presence of fecal loading at the ceacum is a valuable radiologic marker for the diagnosis of acute appendicitis [17]. Petroianu et al. took four groups of patients presenting with flank pain, they divided the patients into four groups; acute appendicitis, urinary calculi, acute gynecologic inflammatory disease, and acute biliary inflammation, the authors reported that fecal loading at the caecum has higher sensitivity of 97.05% for the diagnosis of acute appendicitis, while the sensitivity for urinary calculi was 19.0%, 12% for gynecological inflammatory disease, and only 13% for biliary inflammatory diseases. In this study, the authors determined the diagnostic accuracy of fecal loading at the caecum in diagnosing acute appendicitis, the sensitivity was 98.6% and specificity was 83.3%, which is almost similar to Petrianu et al [17]. While a study by Graham and Johnson reported that X-ray findings can accurately diagnose acute appendicitis (gangrenous or perforated) in only 62% of patients [18]. Their reported accuracy was much lower in comparison to the present study and the above-mentioned studies. 

The present study is limited by the undiversified sample population and recommends that further studies should be conducted using a multicenter approach. In conclusion, on the basis of the results of this study and existing literature we can comment that fecal loading at the caecum is a valuable radiologic marker for the diagnosis of acute appendicitis especially in settings where there is a lack of modern diagnostic tools such as ultrasonography and CT scan.

## Conclusions

According to the results of the present study and existing literature, we suggest using fecal loading at the caecum along with a clinical scoring system for the diagnosis of acute appendicitis. As per our findings, fecal loading at the caecum is a valuable sign-on plain abdominal radiograph for the diagnosis of acute appendicitis. It has a sensitivity of 98.6% and a specificity of 83.3%. This sign typically becomes undetectable after an appendectomy. It will help to improve the accuracy of diagnosis of acute appendicitis, and hence will reduce the chances of negative appendectomy.
